# VOC Profiles of Saliva in Assessment of Halitosis and Submandibular Abscesses Using HS-SPME-GC/MS Technique

**DOI:** 10.3390/molecules24162977

**Published:** 2019-08-16

**Authors:** Fernanda Monedeiro, Maciej Milanowski, Ileana-Andreea Ratiu, Hubert Zmysłowski, Tomasz Ligor, Bogusław Buszewski

**Affiliations:** 1 Department of Environmental Chemistry and Bioanalytics, Faculty of Chemistry, Nicolaus Copernicus University, 7 Gagarina Str., 87-100 Toruń, Poland; 2 Interdisciplinary Centre of Modern Technologies, Nicolaus Copernicus University, 4 Wileńska Str., 87-100 Toruń, Poland; 3 Faculty of Chemistry and Chemical Engineering, Babeş-Bolyai University, 11 Arany Janos, RO-400028 Cluj-Napoca, Romania; 4 Clinical Department of Maxillofacial Surgery in Regional Dental Centre, Located in Provincial Polyclinical Hospital in Toruń, 42 Konstytucji 3 Maja, 87-100 Toruń, Poland

**Keywords:** saliva, halitosis, submandibular abscess, VOCs, GC/MS, diagnosis

## Abstract

Halitosis and submandibular abscesses are examples of mouth-related diseases with the possible bacterial origin. Salivary volatile organic compounds (VOCs) are potential biomarkers of them, once they can be addressed as metabolites of bacterial activity. Healthy patients (n = 15), subjects with submandibular abscesses located in fascial deep space (n = 10), and subjects with halitosis (n = 5) were enrolled in the study. Saliva samples were subjected to headspace solid-phase microextraction (HS-SPME) and gas chromatography coupled to mass spectrometry (GC/MS) analysis. A total number of 164 VOCs was detected by the developed methodology, 23 specific for halitosis and 41 for abscess. Halitosis’ profiles were characterized by a larger number of sulfur compounds, while for abscess they had a higher variety of alcohols, aldehydes, and hydrocarbons—biomarkers of inflammatory processes. Principal components analysis allowed visualization of clusters formed according to the evaluated conditions. Kruskal-Wallis test indicated that 39 VOCs presented differentiated responses between the studied groups, with statistical relevance (p < 0.05). Random forest was applied, and a prediction model based on eight VOCs (2-butanone, methyl thioacetate, 2-methylbutanoic acid, S-methyl pentanethioate, dimethyl tetrasulfide, indolizine, pentadecane, and octadecanal) provided 100% of sensitivity, 82% of specificity, and 91% of balanced accuracy, indicating the specific presence of submandibular abscess.

## 1. Introduction

Halitosis is an oral health condition characterized by unpleasant odor emanating from the oral cavity. At least 50% of the worldwide population is considered as having chronic oral malodor, and 25% of people suffer from discomfort and embarrassment, making halitosis a current and persistent health care problem [1]. This dental issue can have intra-oral and/or extra-oral origin. The main causes are the presence of food deposits and biofilm buildup on the teeth and tongue, resulting from, e.g., poor oral hygiene, improper cleaning of dentures, or decreased salivary flow rate [1,2]. Microorganisms present in the oral cavity, mainly on the dorsum of the tongue, are responsible for 80%–90% of the cases [3]. These are mainly Gram-negative bacteria species, such as *Porphyromonas gingivalis*, *Prevotella intermedia*, *Treponema denticola,* and *Fusobacterium nucleatum* [1]. Malodorous substances are produced by microbial putrefaction of various sulfur-containing substrates like food remnants, components of saliva (especially sulfur-containing amino acids), blood, and epithelial cells [1,3]. To assess halitosis, several techniques are commonly applied, such as organoleptic measurement, sulfide monitoring, chemical sensors, and the benzoyl-DL-arginine-naphthylamide (BANA) test. Organoleptic measurement is the oldest way to assess this disease in sniffing the exhaled air of the mouth and nose from the patient by examiner. Sulfide monitor is a portable device equipped with a disposable tube inserted into the patient’s mouth to collect mouth air. The electrochemical reaction is generated by the presence of volatile sulfur-containing compounds only. Hence, organoleptic measurement enables to detect other odiferous volatiles like alcohols, phenyl compounds, alkenes, ketones, polyamines, and short-chain fatty acids but with difficulties concerning calibration of practitioner and uncomfortable measurement. Chemical sensors are similar in construction and working to sulfide monitors and they can measure each volatile sulfur-containing compounds separately, as well as other volatiles like ammonia. The examined areas of the patient’s mouth are periodontal pockets and the tongue surface. The BANA test detects short-chain fatty acids and proteolytic obligate Gram-negative anaerobes, which hydrolyze the synthetic trypsin substrate and cause halitosis. If *T. denticola*, *P. gingivalis*, or *Bacteroides forsythus* are present in incubated cotton swab sampled from the tongue, the test strip turns blue or the bluer. The method used in this work is a combination of gas chromatography technique and salivary incubation test, a solution developed in 2003. Nowadays, gas chromatography is often used to assess halitosis [4]. The common method involves sampling of breath from a patient using a gas-tight syringe. The subject closes the mouth and holds air for 30 s. Then, mouth air (10 mL) is aspirated and injected into the gas chromatograph column at 70 °C [5]. Salivary incubation test is the name of a technique described in the work of Marc Quirynen et al. 2003. The method assumes the collection of oral fluid in a glass tube and then incubating the tube at 37 °C in an anaerobic chamber under an atmosphere of 80% nitrogen, 10% carbon dioxide, and 10% hydrogen for 3–6 h. After incubation, the odor from tubes was assessed both organoleptically and using the portable sulfide monitor. Volatile sulfur compounds (VSCs) levels were strongly correlated with the organoleptic measurement. Authors concluded that this test could be employed to investigate antimalodor effectiveness of oral hygiene products [6].

Bacterial infections located in the mouth, mainly in the dental area, can lead to the development of oral abscesses [4,7,8,9]. Submandibular abscesses can be caused by submandibular gland sialadenitis, lymphadenitis, trauma, or surgery. The submandibular area comprehends the deep fascial space. A visual inspection may be insufficient to detect an infection in this case; such condition also can be frequently accompanied by subtle clinical manifestations, hindering a proper diagnosis [10]. Also, this may contribute to the development of infections in other regions, such as deep neck spaces. An example is Ludwig’s angina, which is potentially the life-threatening type of severe cellulitis involving the floor of the mouth [11]. The treatment of submandibular space abscesses and Ludwig’s angina consists of open surgical incision and drainage [12]. Bacteria involved in odontogenic infection include *Streptococci*, *Staphylococci*, *Prevotella*, *Peptostreptococcus,* and *Bacteroides* [13]. Bacteria, during metabolic and catabolic processes, emit volatile organic compounds (VOCs), which can, therefore, be used in the assessment of infectious diseases. These compounds, once generated, can be distributed in biological fluids and body compartments. In this sense, saliva emerges as an interesting matrix in the investigation of the mouth-related condition by its physiological connection with craniofacial space. Volatiles in saliva were previously described as potential indicators of other ailments, such as periodontal disease (hydrogen sulfide, pyridine), lung cancer (benzophenone, trans-caryophyllene), and celiac disease (nonanal, 2-hexanone, ethyl acetate) [14,15]. Saliva as a matrix represents a source of VOCs with less complex composition, compared with other matrices. It is easy to collect and store, becoming ideal for early disease detection studies, since it may contain specific biomarkers [14,16].

Consequently, saliva can be useful in the development of non-invasive diagnostic tools, which can provide monitoring of both disease progression and effects of treatments [16,17,18]. Generally, volatiles from bacteria can be preconcentrated by headspace solid-phase microextraction and analyzed by gas chromatography/mass spectrometry (HS-SPME-GC/MS) to detect and identify biomarkers of the presence of bacterial species [19,20,21].

The current study aimed to find potential salivary biomarkers for submandibular abscesses and halitosis, capable of providing a distinction between diseases, through modification and improvement of current methods employed to assess oral malodor. In other words, HS-SPME-GC/MS method combined with salivary incubation test was employed for the first time to examine saliva samples from patients with halitosis and abscesses and control individuals. Moreover, our work modified and improved this method by simplification of sample collection and incubation process and introduction of HS-SPME-GC/MS method, enabling precise and reliable measurements.

## 2. Results and Discussion

### 2.1. Possible Cause and Origin of Detected Volatiles

**Figure 1:**
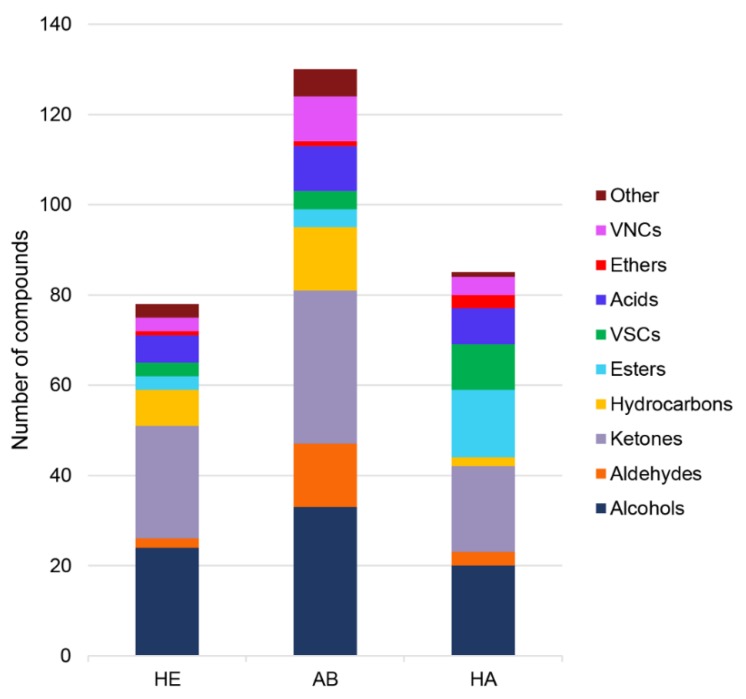
Functional group distribution of VOCs (volatile organic compounds) for healthy patients (HE), subjects with submandibular abscesses (AB), and halitosis (HA), where: VNCs—volatile nitrogen compounds, VSCs—volatile sulfur compounds.

Preliminary experiments (item 1.4 of Supplementary Materials) suggested that incubated samples were superior to “fresh” ones because detected compounds displayed greater signal intensity in this case. It is supposed that the 1-day incubation process, at body temperature, may favor bacterial activity, then, selectively enhancing the content of bacterial volatiles. Incubated samples also allowed better discrimination between samples’ group. The total number of detected VOCs in samples was 164 (Figure 1). The largest number of volatiles (130) was found for patients with submandibular abscess, followed by halitosis sufferers (85) and, finally, healthy volunteers (78).

Evaluation of Figure 1 led to general observations. First, the most predominant classes of compounds for three investigated groups were alcohols and ketones, corresponding, respectively, to 23.2% and 23.8% of the total number of detected VOCs. This finding complied with previous publications regarding salivary volatile profiles [22,23]. Esters corresponded to 9.8%, hydrocarbons to 9.1%, aldehydes to 8.5%, acids and volatile nitrogen compounds (VNCs) to 6.7%, volatile sulfur compounds (VSCs) to 6.1%, ethers to 1.8%, and “others” to 4.3% of the volatiles found in saliva samples. Few compounds were ubiquitous for all three groups of patients, such as 2-methyl-1-propanol, 1-pentanol, 1-dodecanol, 2-heptanone, 6-methyl-5-hepten-2-one, and 2-tetradecanone. These volatiles were commonly found by previous investigators [24,25].

The “abscess” (AB) profiles were characterized by increased number of VNCs (10 of the detected volatiles), hydrocarbons (14 of the detected VOCs), and aldehydes (14 among the total VOCs). Five VNCs were present just in AB profiles: 2,6-dimethylpyrazine, 2,3-dimethylpyrazine, 3-ethenylpyridine, *N*-furfurylpyrrole, and benzyl nitrile. Pyrazines derivatives can origin from bacterial activity as well as they are important flavoring components [26]. 3-ethenylpyridine is a compound considered as an environmental tobacco smoke marker [27,28]. *N*-furfurylpyrrole is found in various foods, such as coffee, chocolate, popcorn, and roasted chicken [29]. Benzyl nitrile (also known as phenylacetonitrile) can be biochemically synthesized by bacterial aldoxime-nitrile pathway, reported in *Escherichia coli* [30] species. Increased number of hydrocarbons can be due to microbial production since bacteria are the main cause of abscesses in mouth. Alkanes are addressed as products of metabolic and catabolic routes of bacterial fatty acids pathway [26]. *Z*-ocimene and myrcene are monoterpenes that, despite being ingredients for the perfumery industry, can be generated by the mevalonate pathway in bacteria [31,32]. Inflammation is often connected to the formation of skin abscesses as a reaction to the infectious process, i.e., the presence of bacteria or parasites. Volatiles representative for inflammatory processes are often compounds generated during oxidative processes and specific for this condition like nitric oxide (NO) and nitrate [33]. Thus, the increased number of aldehydes in AB samples can be considered as a manifestation of local inflammatory processes. Ten aldehydes were specific for the abscess group. Monofunctional C_3_–C_10_ aldehydes are generated as break down products from unsaturated fatty acids after the attack of reactive oxygen species (ROS) onto membrane structures inducing peroxygenation. Hexanal and octanal are examples of such biomarkers [34]. Regarding other aldehydes, 2-methylbutanal and 3-methylbutanal are end-products of protein oxidation occurring in cell structure damage induced by reactive oxygen species [35]. Tetradecanal levels are elevated in exhaled breath from patients with ventilator-associated pneumonia (VAP) in comparison to healthy individuals [36]. Benzaldehyde is a compound found in saliva, hair, and fingernails samples [37]; however, its origin can be exogenous, including gasoline vehicle emissions [38]. (E)-2-octenal and (E)-2-nonenal are expected to be biomarkers of inflammation and/or other carcinogenesis associated processes since they are identified in the headspace of human epithelial cervical carcinoma cells [39]. (E)-2-nonenal is also found as the cucumber odor in the cucumber/farinaceous subgroup of mushrooms with farinaceous odors [40].

In the case of “halitosis” (HA) group, the profiles were characterized by an increased number of volatile sulfur compounds (6 VOCs) and esters (11 VOCs). Predominantly, esters are fatty acid derivatives (released from bacteria), exhibiting a distinctly increased volatility compared to their parent fatty acids [26]. Among them, the most notable were methyl dodecanoate, isopropyl tetradecanoate, 2-ethylhexyl decanoate, and methyl octadecanoate. Volatile sulfur compounds are the most important biomarkers of halitosis detected in breath and saliva [1,41,42]. They are produced by bacteria located in the mouth, such as *P. gingivalis*, *Solobacterium moorei*, *P. intermedia*, *F. nucleatum,* and *T. denticola* [1,7,43]. Sulfur-containing amino-acids, such as cysteine, cysteine, and methionine, are the main substrates yielding VSCs found in saliva or gingival fluid. Three VSCs (hydrogen sulfide, methyl mercaptan, and dimethyl sulfide) are the most referred biomarkers of bad breath in literature [1,3,4]. In our work, we found 10 VSCs occurred in the samples of patients with halitosis. However, the distribution of VSCs among the three investigated groups is presented in Table 1.

Three volatiles, dimethyl disulfide, dimethyl trisulfide, and dimethyl sulfone, were present in control (HE) and diseased groups (AB and HA). Exclusive sulfur-containing compounds for halitosis group were methyl thiolacetate, dimethyl pentasulfide, allyl thiocyanate, allyl isothiocyanate, S-methyl pentanethioate, and thiolan-2-one. These compounds could be potential disease biomarkers and were generated by bacteria incubated from saliva in “limited” (by closed seals of headspace vials) aerobic conditions that allow the growth of predominantly aerobic bacteria or even anaerobic species. Our work modified and improved the salivary incubation test with the incorporation of HS-SPME-GC/MS, instead of organoleptic examination and usage of portable sulfide monitor. The main advantages of the proposed method were high sensitivity and that it could detect a wider spectrum of VOCs at low concentration compared to organoleptic measurement and usage of chemical sensors and portable sulfide monitors. This technique was also highly objective, reproducible, and reliable. Moreover, incubation of saliva gives samples less influenced by food, smoking, and scented cosmetics compared to the gas chromatography technique used alone for the analysis of exhaled “fresh” breath. The disadvantages of the method were time-consuming analyses and the requirement of being carried out by a skilled operator. The costs of GC/MS apparatus could not also be neglected. On the other hand, these drawbacks could be diminished by the high sensitivity of measurements and potential capability to investigate and incorporate new indicators of other dysfunctions and diseases as targets. This methodology also allowed to assess several biomarkers in a single chromatographic run, enabling personalized diagnosis in a sole assay.

Saliva collection protocols may also alter the chemical composition of saliva; hence, the important issue is to maintain high standards of sampling. The procedures of the collection of oral fluid involve non-stimulated (draining, spitting, suction, and adsorption into swab) and stimulated (with chemical or masticatory stimulus) techniques. The use of stimulus, salivary flow rate, and pH are factors that proved to influence the levels of chemical species, such as warfarin, urate, lactate, α-amylase, and cortisol [44,45,46]. In our work, we applied the spitting method to obtain samples with 14-fold more bacteria in specimens than those from passive drooling [47]. This approach indicated effectiveness, considering the presented purpose, once many compounds addressed as bacterial metabolites could be evaluated.

### 2.2. Distribution of Detected VOCs among the Investigated Groups

**Figure 2:**
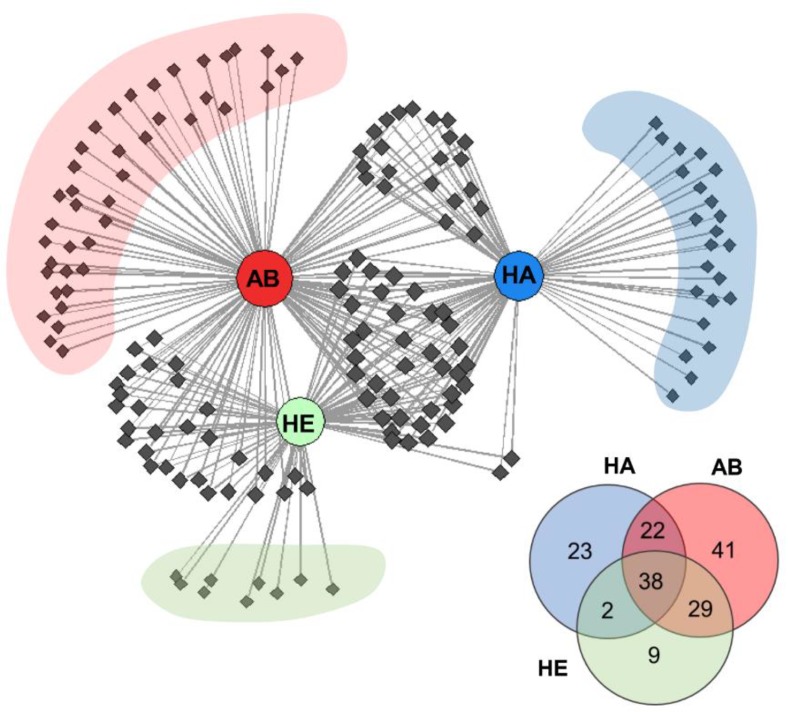
Correlation networks of VOCs emitted from saliva samples from all three group of patients, where: healthy patients (HE), subjects with submandibular abscesses (AB) and halitosis (HA). VOCs are shown as nodes. Numbers in circles denote the number of volatiles from each group.

Figure 2 presents a network analysis graph showing the VOCs distribution for all three investigated groups. For each group, we observed distinctive volatiles present only for healthy (9), abscess (41), and halitosis (23) profiles. It could be noticed that halitosis and healthy groups differed the most—only two compounds were common for them. Abscess group had 22 and 29 VOCs common with halitosis and healthy groups, respectively. There were 38 volatiles common for all three investigated profiles.

### 2.3. Statistical Evaluation and Discrimination of VOC Profiles

**Figure 3:**
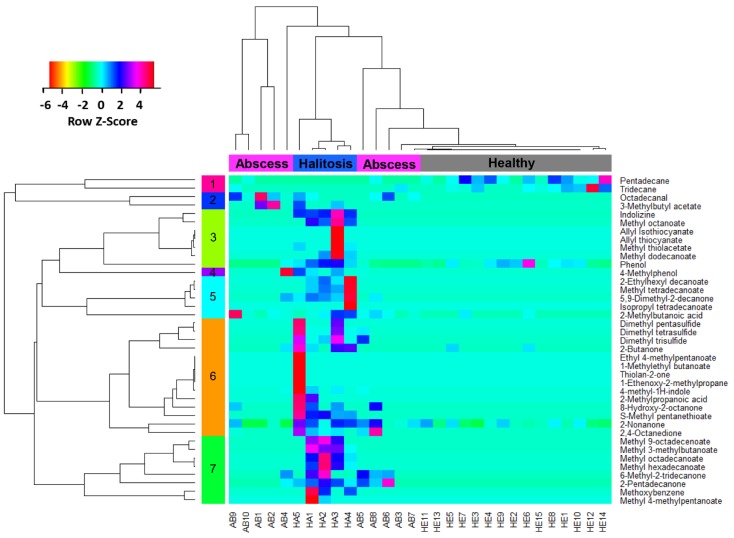
Hierarchical cluster analysis and heatmap of VOC profiles based on peak areas from chromatograms. Columns represent three groups of patients (total of 30 individuals): healthy (15), segregated from an abscess (10) and halitosis (5). Rows represent 39 discriminating VOCs (orange, low abundance; red, high abundance).

Calculated Pearson’s coefficients were all above 0.9 for samples belonging to the same individual, demonstrating high consistency of obtained profiles. Kruskal-Wallis test indicated that **39** VOCs displayed discriminating features when comparing the integrated peak area of compounds from the three studied groups, being the most responsible for obtaining distinct and characteristic HE, AB, and HA profiles. In Figure 3, a heatmap is presented associated with hierarchical cluster analysis using Pearson’s method, which displays the mentioned relevant VOCs, exhibiting the changes in their levels according to analyzed profiles. From the heatmap plot, seven clusters of VOCs were outlined, which were distributed between four clusters formed for the three investigated groups. The abscess group presented two distinct clustering patterns.

The heatmap presented in Figure 3 showed that there were strong links between the chemical structure of VOCs and clusters’ formation. For example, cluster **1** was formed just from hydrocarbons, cluster **3** and **6** were abundant in VSCs, and, in clusters **5** and **7**, esters and methyl-esters, respectively, were predominant.

**Figure 4:**
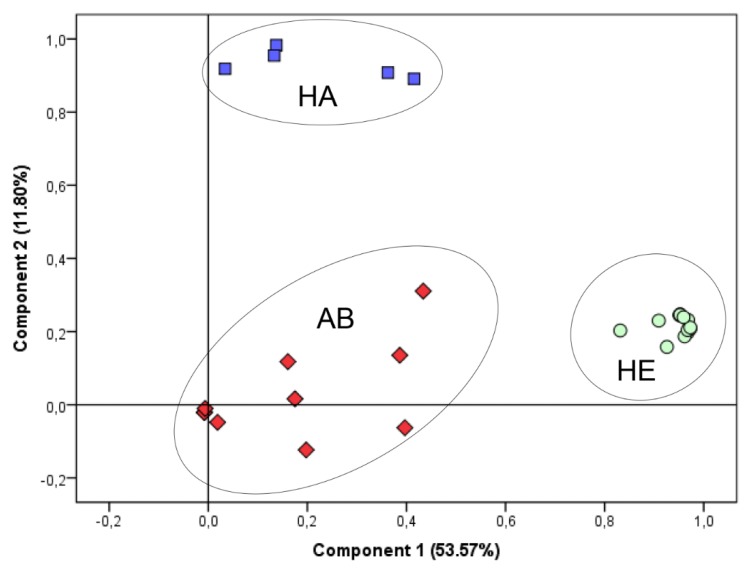
PCA (Principal Component Analysis) plot depicting the differentiation of group of individuals by VOC profiles; Assigned clusters: healthy controls (HE), patients with submandibular abscess (AB) and halitosis (HA).

In the second part of discrimination assessment, the full dataset profiles of detected VOCs were used as input in Principal Components Analysis (Figure 4). The three groups of samples (HA, HE, and AB) formed well-defined clusters, indicating the existence of characteristic VOC profiles associated with each investigated group. PCA plot demonstrated the discrimination based on volatile patterns, where 65.37% of the variance was described for the first two principal components.

### 2.4. Diagnosis Prediction Based on Random Forest Model

**Figure 5:**
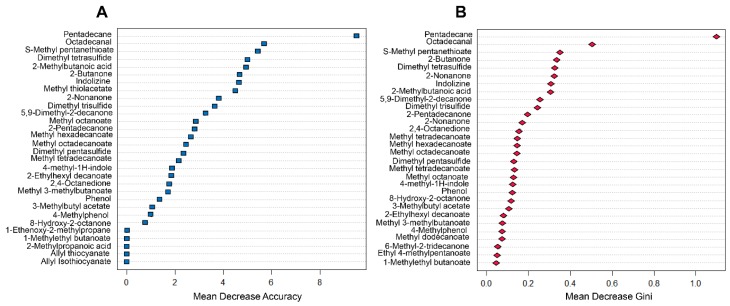
Assessment of variable importance, in terms of (**A**) mean decrease in accuracy and (**B**) mean decrease Gini or node impurity.

**Figure 6:**
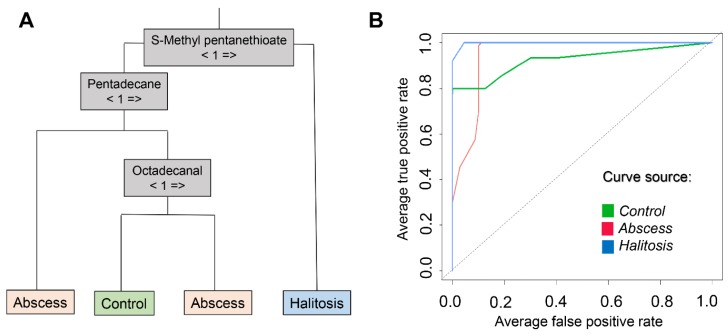
(**A**) Exemplary decision tree produced in Random Forest approach; (**B**) Overlaid ROC (receiver operating characteristic) curves concerning discrimination of specific condition (in green- control / “healthy”, in red- abscess, and in blue- halitosis) against all others.

Random Forest was employed for the development of a classificatory model, able to distinguish controls, halitosis, and abscess cases, based on pattern recognition of the detected volatiles. Initially, the 39 discriminating features posteriorly mentioned were included in this method, and the default number of 500 trees was generated. Figure 5 presents graphs used to assess the importance of variables to the preliminary model. Figure 5A shows the plot of mean decrease accuracy pertinent to each variable, which represents the impact on the prediction ability of the model, in case of exclusion of a given variable. Figure 5B is a plot in terms of mean decrease Gini, related to the relevance of the features in the purity of classes, thus indicating the compounds that are determinant to divide the data into pure nodes, in which the elements belong to a single class. In accordance to these observations, eight compounds addressed as the most relevant for model performance (namely: 2-butanone, methyl thioacetate, 2-methylbutanoic acid, S-methyl pentanethioate, dimethyl tetrasulfide, indolizine, pentadecane, and octadecanal) were selected, and a new Random Forest was performed. The data was split in half, 50% dedicated to training the approach, and 50% applied for model validation step. The obtained “out of bag” score was 6.67%. Figure 6A shows an example of a produced decision tree, in which classification accuracy was 100%. In Figure 6B, overlaid receiver operating characteristic (ROC) curves produced from calculated probabilities in Random Forest model are presented, and they are related to the provided discrimination of each particular condition against all other cases. The results of the performance of this procedure and obtained areas under the curves (AUCs) are presented in Table 2.

## 3. Materials and Methods

### 3.1. Instruments

The GC/MS analyses were carried out using an Agilent 6890A gas chromatograph coupled to an Agilent 5975 Inert XL MSD mass spectrometer (both from Agilent Technologies, Santa Clara, CA, USA). The system was equipped with a Rtx®-5MS w/Integra Guard 30 m × 0.25 mm × 0.25 µm column (Restek Corporation, Bellefonte, PA, USA). Extractions of volatile organic compounds were performed using 65 µm polydimethylsiloxane (PDMS)/divinylbenzene (DVB) fiber (Supelco, Bellefonte, PA, USA).

### 3.2. Materials

Sterile polypropylene tubes (5 mL) packed separately were used for the collection of oral fluid (Eppendorf, Hamburg, Germany). Headspace screw top 20 mL clear vials and magnetic polytetrafluoroethylene (PTFE)/Sil screw caps for headspace vials, 18 mm thread, were purchased from Agilent Technologies (Santa Clara, CA, USA).

### 3.3. Collections of Saliva Samples

The study subjects were adult patients from the Clinical Department of Maxillofacial Surgery in Regional Dental Center, located in Provincial Polyclinical Hospital in Toruń. The Ethical Committee from the Nicolaus Copernicus University of Toruń, Poland, approved the studies. The group of patients was 12 females (F) and 18 males (M) aged between 25 and 65. Fifteen were considered healthy (7F, 8M) and the same amount diseased: 10 with submandibular abscesses (2F, 8M), and 5 with halitosis (3F, 2M). Samples were taken in a non-stimulated manner by saliva ejection into a sterile tube. Sample collection was performed to minimize alterations in the endogenous composition of saliva and to inhibit the addition of interferences from accessory materials.

The participants donated approximately 1.5 mL of saliva, and the collections were performed during the sample period of the day (8:00–11:00 A.M.), in the presence of the physician. No one of the subjects had any dietary restrictions. Participants were instructed to abstain from using chewing gum, eating, and drinking anything except water at least 1 h before sampling. Saliva samples were immediately labeled, transported to the laboratory in a portable thermal container, and stored at −20 °C in the freezer until subsequent thawing and analysis using GC/MS system.

### 3.4. HS-SPME-GC/MS Method

Saliva (0.5 mL) was aliquoted to headspace vials and incubated at 37 °C for 24 h. VOCs were extracted using 65 µm PDMS/DVB fiber at 37 °C for 45 min. All GC/MS experiments were done in triplicate. Carrier gas (helium purity 6.0) flow rate was kept at 1.1 mL min^−1^, and the temperature of injector was set at 240 °C. The oven temperature program was as follows: the initial temperature of 40 °C was kept for 3 min, then ramped at 10 °C min^−1^ to 300 °C and kept for 5 min. Spectra acquisition was performed within a range of 30–300 *m/z*, in electron ionization (EI) mode, at 70 eV; both the ion source and the transfer line temperature was set at 250 °C. Compounds were identified by comparing their mass spectra with those contained in the NIST mass spectral library version 2005; each peak was searched manually (including baseline subtraction and averaging over a peak). Forward and reverse match quality of at least 800/1000 was used as the lower match threshold; otherwise, a compound was labeled as unknown.

Experiments regarding the optimization of the methodology are detailed in Item 1 of Supplementary Materials, including the influence of fiber selection, time of extraction, sample volume, and saliva incubation. Although the present work comprised a non-target approach, an internal validation process was performed to certify the suitability of the method. Saliva samples and blank samples spiked with standards of model VOCs were employed. The assays evaluated limits of detection and quantitation, linearity, accuracy, precision, stability, and matrix effect (Item 2 of Supplementary Materials). The results showed that the evaluated parameters met the criteria that indicate the reliability of the method.

### 3.5. Statistical Approaches

Statistical analyses were performed using IBM SPSS Statistics v. 42. Pearson’s correlation analysis was performed to verify the variability between samples triplicates from the same individual. The remaining data analysis was made considering an average of these replicated profiles. Kruskal-Wallis and Mann-Whitney tests were applied to indicate the compounds which presented statistically relevant differences in their responses in the studied subjects’ group, considering conventional relevance criteria of p < 0.05. Principal components analysis (PCA) was executed to visualize discrimination between VOCs profiles from samples of patients.

The following methods were processed in R environment, using RStudio console v. 1.1.463 and employing the packages: “gplots”, “sna”, “randomForest”, “caret”, and “ROCR”. Heatmap associated with hierarchical cluster analysis using Pearson’s clustering method was built to provide visualization of changes in the intensity of compounds responses. Network analysis was created to exhibit links between detected compounds and the groups originating them. For evaluation of a classificatory model, Random Forest was employed. This machine-learning algorithm is based on the combination of multiple decision trees, resulting in an improved training model, which can provide discrimination between the categories of pattern’s sources accordingly to a system of pattern recognition. In a first step, the relative importance of each feature to the prediction model was assessed to select a smaller number of features for the design of the simpler final model. Then, in the form of cross-validation, the trained model was applied to an unknown set of samples (“out of bag” samples), and the results of the model’s performance were evaluated.

For the elaboration of heatmap and hierarchical cluster analysis, data standardization by Z-score normalization was carried out. For network analysis, the peak database was converted into binary entries, where 0 means absence and 1, presence. For Random Forest, binary features were also employed.

## 4. Conclusions

The analyses of volatile organic compounds proved to be a useful analytical tool to assess mouth-related bacterial conditions. Our work focused on the development of a new methodology to differentiate oral malodor from the presence of a submandibular abscess, a condition not always easily recognized by conventional medical examination. Volatiles from halitosis cohort were abundant in sulfur-containing compounds originated from bacterial metabolism and also esters. Submandibular abscess group was characterized by the occurrence of inflammation indicators, like aldehydes. Statistical approaches used in our research showed that the combination of saliva incubation and GC/MS analysis is a promising method for discrimination of patients with halitosis, abscess, and those presenting healthy oral microbiota. In a future perspective, the developed method can be employed in investigations regarding which bacterial strains contribute the most to disease conditions and whether it is possible to find differences in VOC profiles from other types of skin abscesses. Moreover, the present study could represent a valuable material for companies interested to build or calibrate dedicated sensors, which have the ability for real-time detection of these kinds of diseases based on some targeted components.

## Figures and Tables

**Table 1:** List of volatile sulfur compounds detected in incubated salivary samples from healthy patients (HE) and subjects with submandibular abscesses (AB) and halitosis (HA); “X” means the presence of the compound.

Volatile Sulfur Compound	Group of Patients		
	HE	AB	HA
methyl thiolacetate			X
dimethyl disulfide	X	X	X
dimethyl trisulfide	X	X	X
dimethyl tetrasulfide		X	X
dimethyl pentasulfide			X
dimethyl sulfone	X	X	X
allyl thiocyanate			X
allyl isothiocyanate			X
S-methyl pentanethioate			X
thiolan-2-one			X
TOTAL	3	4	10

**Table 2:** Sensitivity, specificity, balanced accuracy, and AUC (area under the curve) obtained from Random Forest-based model for classification of studied clinical conditions.

Class	Control	Submandibular Abscess	Halitosis
Sensitivity	66.7%	100.0%	100.0%
Specificity	100%	81.8%	92.3%
Balanced accuracy	83.3%	90.9%	96.1%
AUC	0.927	0.950	0.992

## Supplementary Materials

The following are available online, Figure S1: Relative recovery (%) of detected compounds for different SPME fiber’s coatings (sample volume = 1 mL, extraction time = 45 min, extraction temperature = 37 °C), Figure S2: Average total area obtained for different times of extraction (sample volume = 1 mL, extraction temperature = 37 °C), Figure S3: Average total area obtained for different volumes of samples, number of peaks is presented above the bars (extraction time = 45 min, extraction temperature = 37 °C), Figure S4: Comparison between fresh and incubated saliva, in terms of (A) average total area and (B) number of detected peaks, Table S1: Model analytes, limit of detection, limit of quantification, defined quality controls and linearity data, Table S2: Average imprecision calculated between triplicates of real samples (RSD% = relative standard deviation), Table S3: Average imprecision and inaccuracy calculated for spiked samples (RSD% = relative standard deviation, RSE% = relative standard error), Table S4: Stability results obtained from saliva pool samples, Table S5: Stability results obtained from spiked samples, Table S6: Matrix effect (NMF = normalized matrix factor, RSD% = relative standard error).
