# Supplementary material for: VOC Profiles of Saliva in Assessment of Halitosis and Submandibular Abscesses Using HS-SPME-GC/MS Technique
Source: Molecules. 2019 Aug 16;24(16):2977. doi: 10.3390/molecules24162977 (PMC6720996; doi:10.3390/molecules24162977)
Supplement: Supplementary file 1 [file molecules-24-02977-s001.pdf]

Supplementary material- “VOC profiles of saliva in assessment of halitosis and submandibular abscesses using HS-SPME-GC/MS technique”

1. Methodology optimization

1.1. Fiber selection (recovery assessment)

The performance of the three most often employed SPME fibers (CAR/PDMS- 75 µm, DVB/PDMS- 65 µm and PDMS-100 µm) was evaluated and results were expressed in terms of relative response. Pool of saliva samples belonging to 6 different control individuals were evaluated in triplicate, with each model of fiber. Figure 1 presents the obtained results, showing that a best recovery for most of the compounds was obtained when DVB/PDMS fiber was employed, being that the fiber selected for the further experiments.

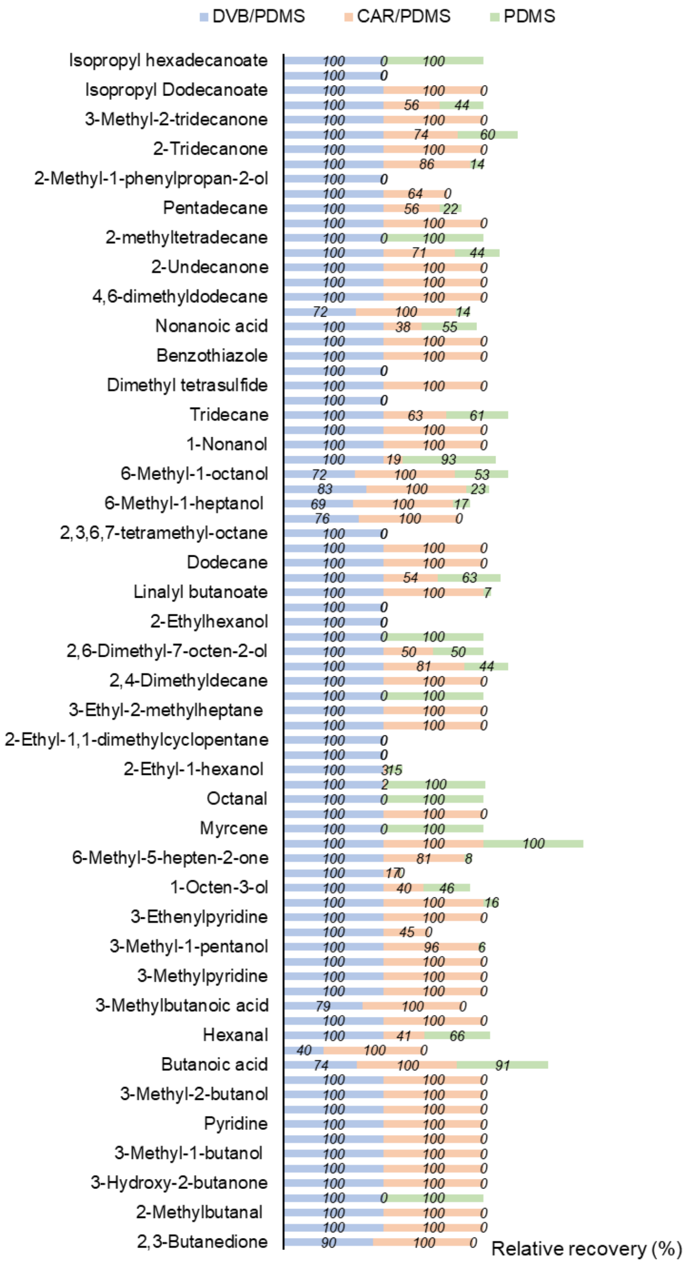

Figure 1- Relative recovery (%) of detected compounds for different SPME fiber’s coatings (sample volume = 1 mL, extraction time = 45 min, extraction temperature = 37°C).

1.2. Extraction time

Different extraction times (10, 30, 45 and 60 min) were tested in triplicate for a pool of saliva, prepared as described in 1.1.. A graph depicted in Figure 2 presents obtained average total area (summed up areas of detected peaks) for each time tested. Extraction conducted during 45 min displayed acceptable performance and lower standard deviation was observed, indicating better reproducibility of profiles.

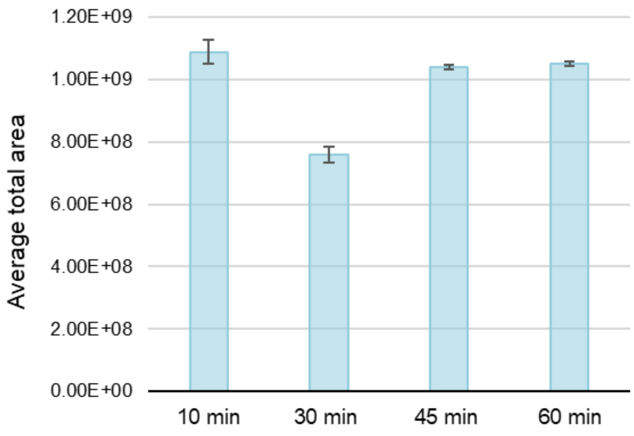

Figure 2- Average total area obtained for different times of extraction (sample volume = 1 mL, extraction temperature = 37°C).

1.3. Sample volume

Different volumes of sample (0.5, 1, 2 and 3 mL) were tested in triplicate for a pool of saliva, prepared as described in 1.1.. A graph depicted in Figure 3 presents obtained average total area (summed up areas of detected peaks) for each tested volume, as well as the average number of detected peaks. 0.5 mL and 1 mL presented to be the most suitable volumes for saliva sample analysis, since larger number of peaks could be detected. Since no analytical disadvantage was verified in the present case, the use of lower amount of biological specimen was prioritized.

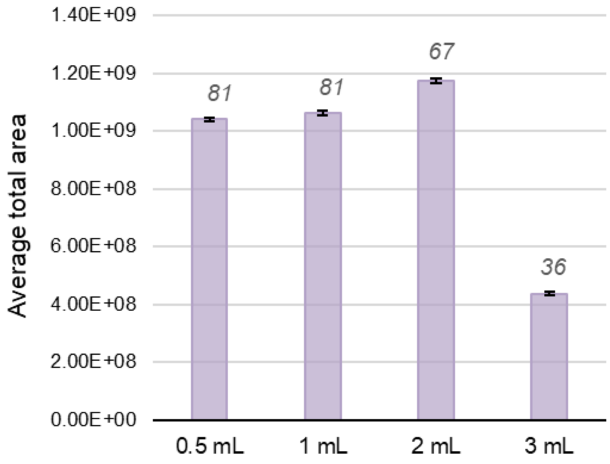

Figure 3- Average total area obtained for different volumes of samples, number of peaks is presented above the bars (extraction time = 45 min, extraction temperature = 37°C).

1.4. Fresh saliva *vs.* incubated saliva

Aliquots of fresh saliva samples were compared to samples submitted to incubation at 37 ℃, for 24 h. These samples correspond to those enrolled in this study (n = 30). The results were expressed in terms of average total area (average of sum of peak areas obtained for a group of samples) and number of detected peaks. The Figure 4 depicts these observations, highlighting the obtaining of richer profiles when incubation step is incorporated.

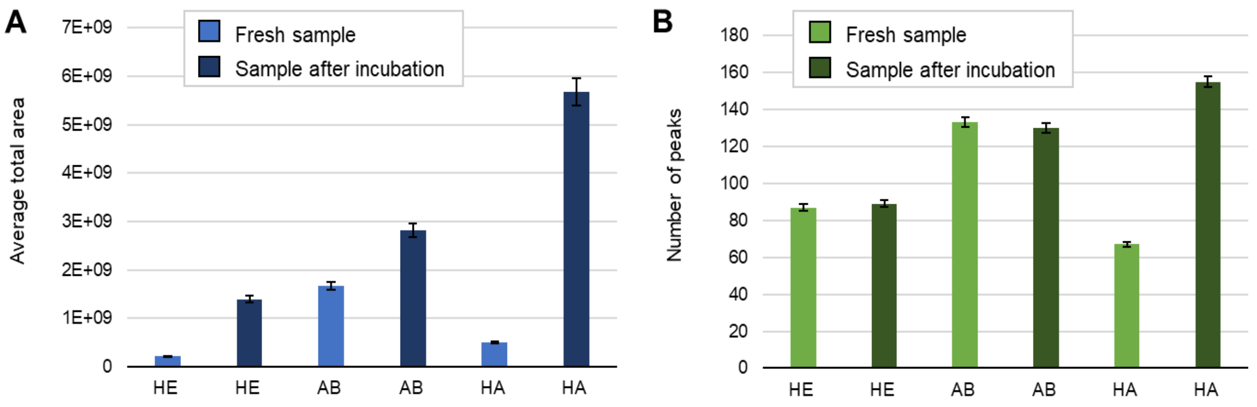

Figure 4- Comparison between fresh and incubated saliva, in terms of (A) average total area and (B) number of detected peaks.

2. Internal validation

In order to verify the suitability of the developed method to its purpose, a simplified validation process was carried out. The following parameters were evaluated: precision, accuracy, linearity, carry over, matrix effect and stability. Once the present work is based in non-target metabolomics and endogenous compounds are being investigated, 26 VOCs standards were used as model analytes and spiked to blank samples (0.5 mL of deionized water – simulating the major content of the biological sample). 4-bromofluorobenzene was used as the internal standard (IS), added to samples at fixed concentration of 100 ng mL^{-1}. The spiked "artificial" samples were processed exactly like the saliva samples (including incubation step, for 24 h). The Table 1 presents the used chemical standards and the concentrations evaluated as the quality control samples (QC). The limit of detection (LOD) was defined as the minimal concentration providing signal-to-noise ratio equal 3, the limit of quantitation (LOQ) was defined as the lowest concentration presenting imprecision and inaccuracy below 15%.

Table 1- Model analytes, limit of detection, limit of quantification, defined quality controls and linearity data

Analyte	LOD	LOQ	QC1	QC2	QC3	Linearity range	Equation	R
	(ng mL^{-1})							
2-Methylpentane	0.01	0.025	0.05	25	50	0.025-75	y = 0.0403x + 0.2324	0.987
3-Methylpentane	0.01	0.025	0.05	25	50	0.025-75	y = 0.0505x + 0.4176	0.985
Methylcyclopentane	0.01	0.025	0.05	25	50	0.025-75	y = 0.0878x + 0.2035	0.986
2-Butanone	1	5	10	125	250	5-300	y = 0.0634x + 5.3276	0.990
Benzene	0.01	0.025	0.05	25	50	0.025-75	y = 0.2512x - 0.3553	0.992
Toluene	0.01	0.025	0.05	25	50	0.025-75	y = 1.2438x + 1.3082	0.994
Ethylbenzene	0.01	0.025	0.05	25	50	0.025-75	y = 3.7318x + 1.7221	0.998
p-Xylene	0.01	0.025	0.05	25	50	0.025-75	y = 9.9641x + 130.42	0.990
Styrene	0.01	0.025	0.05	25	50	0.025-75	y = 7.8678x + 90.561	0.992

Decane	0.01	0.025	0.05	25	50	0.025-75	y = 0.1184x - 0.0518	0.998
6-Methyl-2-heptanone	0.01	0.025	0.05	25	50	0.025-75	y = 0.1008x - 0.0039	0.996
Isododecane	0.01	0.025	0.05	25	50	0.025-75	y = 5.3357x + 4.3964	0.999
1,2,4-Trimethylbenzene	0.01	0.025	0.05	25	50	0.025-75	y = 9.1189x - 4.3529	0.996
E-Ocimene	0.01	0.025	0.05	25	50	0.025-75	y = 2.89x - 2.6382	0.995
Limonene	0.01	0.025	0.05	25	50	0.025-75	y = 1.3739x + 2.1648	0.996
m-Cymene	0.01	0.025	0.05	25	50	0.025-75	y = 3.693x - 5.2705	0.995
Benzonitrile	0.01	0.025	0.05	25	50	0.025-75	y = 0.7995x - 0.2695	0.999
Phenol	1	5	10	125	250	5-300	y = 0.0153x + 0.3864	0.999
Undecane	0.01	0.025	0.05	25	50	0.025-75	y = 6.8805x + 1.6561	0.999
Dodecane	0.01	0.025	0.05	25	50	0.025-75	y = 4.7759x + 0.3487	0.996
Terpineol	0.1	0.25	0.5	125	250	0.25-300	y = 0.0987x + 1.8353	0.987
Tridecane	0.1	0.25	0.5	125	250	0.25-300	y = 0.2761x + 2.3961	0.999
Hexadecane	0.1	0.25	0.5	125	250	0.25-300	y = 0.5317x + 7.7336	0.992
Ethyl-4-nitrobenzoate	0.1	0.25	0.5	125	250	0.25-300	y = 0.0274x + 0.9927	0.998
Octadecane	0.1	0.25	0.5	125	250	0.25-300	y = 0.2619x + 3.8848	0.994
Nonadecane	0.1	0.25	0.5	125	250	0.25-300	y = 0.5954x + 7.8596	0.994

2.1. Precision and accuracy

The precision was evaluated: i) based in the samples analyzed in triplicate (3 aliquots); ii) model analytes spiked in concentrations corresponding to QC1 and QC3. The accuracy was assessed considering the relative standard error (= average experimental concentration – theoretical concentration x 100 / theoretical concentration). Table 2 and Table 3 present the obtained results. The calculated imprecision and inaccuracy did not exceed 15% for the LOQ and 10% for other concentrations.

Table 2- Average imprecision calculated between triplicates of real samples (RSD% = relative standard deviation)

Analyte	RSD%
1-Propanol	5.37
Acetic acid	2.65
2-Butanone	1.95
2,3-Butanedione	2.56
2-Methylpropanal	2.19
2-Methyl-1-propanol	0.35
3-Methylbutanal	6.82
1-Butanol	5.23
2-Methylbutanal	2.62
Methyl thiolacetate	7.94
2,3-Pentanedione	8.22
2-Propanol	5.16
2-Ethoxypropane	1.94
3-Hydroxy-2-butanone	6.55
3-Methyl-3-buten-1-ol	2.85
1-Pentanol	1.77
3-Methyl-1-butanol	2.86
1,2-Dimethylcyclopropane	9.33
1-Ethenoxy-2-methylpropane	8.15
2-Methyl-1-butanol	4.51

4-Methylpentan-2-one	0.60
Dimethyl disulfide	1.73
Dimethyl sulfone	4.77
Pyridine	1.46
3-Methyl-2-pentanone	6.04
2-Methylpropanoic acid	7.74
2-Pentanol	7.76
Methyl 3-methylbutanoate	4.73
3-Methyl-2-butanol	8.14
2-Methyl-3-pentanone	9.61
2,3-Hexanedione	3.53
Butanoic acid	3.75
3-Hexanone	4.41
2,3-Butanediol	4.52
2-Methylpentanal	2.67
Octane	0.17
Hexanal	1.92
3-Pentanol	3.54
2-Hydroxy-3-pentanone	1.56
Butyl propanoate	3.03
1-Hexanol	1.82
2-Methylcyclopentanone	1.06
3-Methylbutanoic acid	7.19
2-Methylbutanoic acid	5.97
2-Furanmethanol	4.35
1-Methylethyl butanoate	0.12
5-Methyl-2-hexanone	7.85
3-Methylpyridine	8.48
2-Methylpropanoic acid	1.01
Allyl thiocyanate	2.53
3-Methyl-1-pentanol	6.04
2-Methyl-3-hexanone	8.78
3-Methylbutyl acetate	3.70
Pentanoic acid	3.70
2,4-Dimethyl-3-pentanone	6.73
Allyl Isothiocyanate	0.40
2,5-Dimethylcyclopentanone	4.66
2-Heptanone	0.03
Styrene	8.52
Methyl 4-methylpentanoate	4.77
2,5-Dimethyl-3-hexanone	3.22
Propyl butanoate	0.51
2,6-Dimethylpyrazine	1.62
Methoxybenzene	1.07
2,3-Dimethylpyrazine	8.31
4-Methyl-2-heptanone	9.16
2-Ethylcyclopentanone	3.78
S-Methyl pentanethioate	1.51
2,6-Dimethyl-4-heptanol	1.12
4-Methylpentanoic acid	8.19

3-Methylcyclohexanone	0.16
Benzaldehyde	4.86
Ethyl 4-methylpentanoate	0.01
3-Ethenylpyridine	4.71
1-Heptanol	9.80
Dimethyl trisulfide	9.78
Acetophenone	3.31
1-Octen-3-ol	8.29
Phenol	8.58
6-Methyl-5-hepten-2-one	5.97
Phenyl carbamate	6.35
5-Methyl-3-heptanone	3.95
2-Octanone	2.73
Myrcene	3.72
2-Pentylfuran	1.35
Octanal	2.16
Thiolan-2-one	7.47
Hexanoic acid	4.03
3,7-Dimethyl-1,3,6-octatriene	4.33
2,4-Octanedione	8.16
6-Methyl-2,4-heptanedione	9.51
o-Cymene	5.41
2-Ethyl-1-hexanol	2.15
Z-Ocimene	8.68
Benzeneacetaldehyde	4.70
3-Ethyl-2-methylheptane	1.55
8-Hydroxy-2-octanone	4.26
E-2-Octenal	0.46
2,4-Dimethyldecane	2.37
E-2-Octen-1-ol	1.63
2,6-Dimethyl-7-octen-2-ol	6.92
Pentylcyclopropane	6.42
4-Methylphenol	5.46
3-Methylphenol	4.50
2-Ethylhexanol	4.41
2-Nonanone	9.96
2-Methoxyphenol	1.28
1-Dodecene	5.53
Linalyl butanoate	4.21
Nonanal	4.89
Phenylethyl Alcohol	2.94
2-Nonanol	3.94
Methyl octanoate	1.78
6-Methyl-1-heptanol	5.73
5-Methyl-1-heptanol	3.15
7-Methyloctane-2,4-dione	8.50
5-Methyl-octanone	5.22
E-2-Nonenal	2.94
2-Decanone	7.82
Octanoic Acid	3.46

1-Nonanol	7.04
N-Furfurylpyrrole	5.77
2,7-Dimethyl-1-octanol	3.33
Tridecane	1.33
Methyl 2-hydroxybenzoate	0.63
Dimethyl tetrasulfide	7.35
3,7-dimethyl-2-octen-1-ol	4.05
Z-3,7-dimethyl-2,6-octadien-1-ol	5.54
Nonanoic acid	8.81
1-Decanol	5.50
4,6-dimethyldodecane	5.73
4-ethyl-2-methoxyphenol	9.33
2-Undecanone	7.92
Indole	0.23
Benzyl nitrile	1.96
Indolizine	1.99
2,4-Decadienal	5.16
5,9-Dimethyl-2-decanone	7.05
2-Dodecanone	4.90
1-Pentadecene	5.94
4-methyl-1H-indole	0.98
Pentadecane	0.70
2-Methyl-1-phenylpropan-2-ol	2.43
5,9-Undecadien-2-one, 6,10-dimethyl-, (Z)-	7.28
2-Tridecanone	7.00
1-Dodecanol	6.73
Dimethyl pentasulfide	7.87
Methyl dodecanoate	0.94
6-Methyl-2-tridecanone	4.35
3-Methyl-2-tridecanone	7.12
2-Tetradecanone	7.49
Tetradecanal	4.70
Isopropyl Dodecanoate	2.85
2-Pentadecanone	9.41
Methyl tetradecanoate	4.76
3,7,11-Trimethyl-1-dodecanol	6.25
2-Hexadecanone	8.64
Hexadecanal	8.36
Isopropyl tetradecanoate	0.12
2-Ethylhexyl decanoate	4.28
Methyl hexadecanoate	2.27
Isopropyl hexadecanoate	5.78
Methyl 9-octadecenoate	6.69
Methyl octadecanoate	5.78

Table 3- Average imprecision and inaccuracy calculated for spiked samples (RSD% = relative standard deviation, RSE% = relative standard error)

**Analyte**	**RSD%**				**RSE%**			
	LOQ	QC1	QC2	QC3	LOQ	QC1	QC2	QC3
2-Methylpentane	13.88	8.60	6.10	5.64	-5.13	-7.60	6.56	-5.31
3-Methylpentane	2.47	8.16	1.44	2.32	6.30	5.25	-8.46	-7.33
Methylcyclopentane	-13.88	4.83	6.07	6.82	4.35	4.93	7.47	8.87
2-Butanone	2.19	6.43	8.30	4.86	-11.85	1.59	5.56	3.58
Benzene	7.62	7.56	8.63	7.47	5.93	-10.35	4.22	4.12
Toluene	3.11	10.91	4.82	4.95	11.68	5.54	5.56	5.65
Ethylbenzene	5.03	1.26	7.65	4.51	3.57	-8.73	7.29	-2.98
p-Xylene	10.46	2.94	8.84	6.31	-4.44	8.28	8.01	1.86
Styrene	3.75	4.97	7.06	7.90	11.29	-7.35	6.67	1.73
Decane	6.41	3.54	3.50	8.04	-8.78	4.22	3.90	4.47
6-Methyl-2-heptanone	7.26	8.87	7.98	4.23	2.63	-7.00	4.71	2.81
Isododecane	9.03	7.74	8.51	7.57	-8.04	-7.07	-2.70	-2.69
1,2,4-Trimethylbenzene	6.83	1.41	5.28	7.68	-5.85	-1.04	8.54	8.71
E-Ocimene	6.43	10.98	8.85	1.78	4.62	10.40	3.65	6.69
Limonene	5.91	3.43	7.35	6.54	11.64	-8.32	-8.12	-6.14
m-Cymene	10.46	4.26	5.55	1.07	8.68	5.26	-6.62	1.04
Benzonitrile	11.22	1.70	7.11	4.39	-11.22	-8.62	-3.34	5.73
Phenol	10.71	3.04	8.81	1.50	-6.88	-8.29	1.77	-4.56
Undecane	10.84	9.00	1.86	7.89	7.92	1.27	2.90	2.94
Dodecane	6.91	3.12	5.00	3.57	1.68	8.60	3.71	4.15
Terpineol	13.48	7.33	5.57	7.26	11.68	1.42	3.77	4.33
Tridecane	9.56	4.70	5.64	7.36	5.69	-10.05	-5.77	1.47
Hexadecane	3.71	8.27	8.63	3.66	1.83	3.44	3.98	6.47
Ethyl-4-nitrobenzoate	12.41	6.91	6.66	3.53	-8.72	-7.15	4.37	-5.58
Octadecane	4.48	7.91	7.63	1.97	4.81	10.62	-7.12	5.80
Nonadecane	9.55	5.03	5.61	2.36	6.53	10.28	2.22	1.05

2.2. Stability

The stability of VOCs in samples was assessed for the following conditions: 24 h, at room temperature (21 ℃) and 2 thaw cycles (freezer at -20 ℃). The evaluation was made in triplicate, for two types of samples: i) processed pool of saliva samples, obtained from collected samples (n = 30), ii) spiked deionized water, at concentration levels corresponding to QC1 and QC3. The internal standard was added just prior analysis. For procedure i), stability was assessed calculating percentual variation of the response in comparison to samples freshly prepared and analyzed. Stability in approach ii) was described in terms of deviation from nominal concentration. The results are displayed in Table 4 and 5. The calculated data register alterations not superior to 15%, thus, sample stability can be attested.

Table 4 -Stability results obtained from saliva pool samples

**Analyte**	**Average deviation from the response in fresh sample (%)**	
	Post-processing stability (24h, 21 ℃)	2 Thaw cycles (-20 ℃)
1-Propanol	-1.93	-9.05
Acetic acid	-4.68	-7.00
2-Butanone	-2.10	-10.34
2,3-Butanedione	-4.46	-7.08
2-Methylpropanal	-1.50	-10.37
2-Methyl-1-propanol	-4.15	-2.23
3-Methylbutanal	-9.30	-6.97
1-Butanol	-3.06	-4.10
2-Methylbutanal	-2.29	-7.43
Methyl thiolacetate	-1.20	-4.00
2,3-Pentanedione	-2.18	-7.21
2-Propanol	-4.24	-1.79
2-Ethoxypropane	-2.88	-1.74
3-Hydroxy-2-butanone	-8.02	-5.08
3-Methyl-3-buten-1-ol	-1.86	-6.97
1-Pentanol	-8.00	-8.11
3-Methyl-1-butanol	-8.82	-10.90
1,2-Dimethylcyclopropane	-7.54	-6.38
1-Ethenoxy-2-methylpropane	-1.94	-2.32
2-Methyl-1-butanol	-5.82	-1.38
4-Methylpentan-2-one	-6.72	-2.77
Dimethyl disulfide	-1.98	-8.86
Dimethyl sulfone	-5.98	-7.37
Pyridine	-1.75	-2.34
3-Methyl-2-pentanone	-6.81	-4.91
2-Methylpropanoic acid	-5.66	-8.02
2-Pentanol	-4.46	-2.27
Methyl 3-methylbutanoate	-3.72	-2.12
3-Methyl-2-butanol	-4.35	-9.46
2-Methyl-3-pentanone	-2.60	-2.35
2,3-Hexanedione	-4.05	-3.94
Butanoic acid	-5.20	-3.96
3-Hexanone	-7.16	-3.67
2,3-Butanediol	-5.87	-2.93
2-Methylpentanal	-7.93	-6.12
Octane	-8.77	-11.00
Hexanal	-3.34	-2.34
3-Pentanol	-6.33	-2.90
2-Hydroxy-3-pentanone	-6.21	-10.99
Butyl propanoate	-9.63	-10.81
1-Hexanol	-6.49	-3.87
2-Methylcyclopentanone	-5.10	-7.71
3-Methylbutanoic acid	-6.44	-8.05

2-Methylbutanoic acid	-4.21	-7.73
2-Furanmethanol	-3.39	-8.12
1-Methylethyl butanoate	-4.19	-7.41
5-Methyl-2-hexanone	-7.52	-9.47
3-Methylpyridine	-6.89	-5.41
2-Methylpropanoic acid	-5.09	-5.87
Allyl thiocyanate	-4.76	-9.96
3-Methyl-1-pentanol	-1.28	-5.25
2-Methyl-3-hexanone	-1.89	-8.41
3-Methylbutyl acetate	-3.35	-9.14
Pentanoic acid	-3.73	-8.40
2,4-Dimethyl-3-pentanone	-7.68	-7.79
Allyl Isothiocyanate	-7.13	-9.36
2,5-Dimethylcyclopentanone	-6.45	-4.96
2-Heptanone	-6.98	-1.93
Styrene	-8.29	-1.91
Methyl 4-methylpentanoate	-1.96	-5.66
2,5-Dimethyl-3-hexanone	-5.07	-7.97
Propyl butanoate	-3.29	-1.53
2,6-Dimethylpyrazine	-1.39	-4.24
Methoxybenzene	-1.63	-7.75
2,3-Dimethylpyrazine	-2.05	-3.60
4-Methyl-2-heptanone	-1.27	-7.69
2-Ethylcyclopentanone	-7.39	-3.59
S-Methyl pentanethioate	-5.51	-6.03
2,6-Dimethyl-4-heptanol	-8.00	-6.11
4-Methylpentanoic acid	-3.73	-3.69
3-Methylcyclohexanone	-8.12	-6.62
Benzaldehyde	-7.87	-7.97
Ethyl 4-methylpentanoate	-1.31	-9.49
3-Ethenylpyridine	-7.76	-7.78
1-Heptanol	-6.18	-8.25
Dimethyl trisulfide	-7.67	-10.28
Acetophenone	-2.37	-2.16
1-Octen-3-ol	-1.04	-10.53
Phenol	-2.14	-10.04
6-Methyl-5-hepten-2-one	-2.71	-9.24
Phenyl carbamate	-4.44	-5.82
5-Methyl-3-heptanone	-2.34	-5.81
2-Octanone	-2.31	-6.90
Myrcene	-7.07	-1.60
2-Pentylfuran	-6.85	-8.02
Octanal	-2.22	-8.11
Thiolan-2-one	-6.81	-8.42
Hexanoic acid	-4.09	-9.00
3,7-Dimethyl-1,3,6-octatriene	-6.90	-2.19
2,4-Octanedione	-3.19	-2.74
6-Methyl-2,4-heptanedione	-6.57	-2.67
o-Cymene	-5.53	-2.68
2-Ethyl-1-hexanol	-5.97	-3.03

Z-Ocimene	-9.48	-9.87
Benzeneacetaldehyde	-9.38	-8.74
3-Ethyl-2-methylheptane	-1.63	-4.31
8-Hydroxy-2-octanone	-3.46	-4.47
E-2-Octenal	-6.78	-10.49
2,4-Dimethyldecane	-2.10	-2.17
E-2-Octen-1-ol	-4.91	-1.57
2,6-Dimethyl-7-octen-2-ol	-8.27	-2.17
Pentylcyclopropane	-8.13	-8.30
4-Methylphenol	-5.10	-6.50
3-Methylphenol	-8.16	-10.06
2-Ethylhexanol	-7.85	-4.35
2-Nonanone	-4.36	-6.86
2-Methoxyphenol	-6.48	-3.49
1-Dodecene	-7.97	-3.78
Linalyl butanoate	-6.06	-2.05
Nonanal	-5.36	-2.60
Phenylethyl Alcohol	-7.08	-7.67
2-Nonanol	-5.83	-9.78
Methyl octanoate	-6.59	-2.33
6-Methyl-1-heptanol	-8.13	-8.19
5-Methyl-1-heptanol	-8.26	-9.05
7-Methyloctane-2,4-dione	-9.28	-4.48
5-Methyl-octanone	-7.18	-5.23
E-2-Nonenal	-9.43	-9.29
2-Decanone	-9.76	-8.16
Octanoic Acid	-4.56	-1.08
1-Nonanol	-1.80	-10.00
N-Furfurylpyrrole	-8.58	-9.67
2,7-Dimethyl-1-octanol	-6.00	-1.98
Tridecane	-9.58	-1.49
Methyl 2-hydroxybenzoate	-5.07	-8.72
Dimethyl tetrasulfide	-4.34	-7.75
3,7-dimethyl-2-octen-1-ol	-5.35	-5.54
Z-3,7-dimethyl-2,6-octadien-1-ol	-9.01	-4.82
Nonanoic acid	-9.98	-3.35
1-Decanol	-8.80	-2.96
4,6-dimethyldodecane	-6.70	-1.06
4-ethyl-2-methoxyphenol	-6.37	-7.59
2-Undecanone	-4.64	-1.23
Indole	-9.42	-1.76
Benzyl nitrile	-9.49	-10.11
Indolizine	-6.60	-5.49
2,4-Decadienal	-9.85	-10.73
5,9-Dimethyl-2-decanone	-9.77	-5.14
2-Dodecanone	-4.01	-3.57
1-Pentadecene	-2.47	-1.14
4-methyl-1H-indole	-5.90	-4.83
Pentadecane	-7.33	-4.11
2-Methyl-1-phenylpropan-2-ol	-5.99	-8.16

5,9-Undecadien-2-one, 6,10-dimethyl-, (Z)-	-1.87	-1.32
2-Tridecanone	-8.71	-7.05
1-Dodecanol	-7.11	-9.05
Dimethyl pentasulfide	-4.53	-7.96
Methyl dodecanoate	-4.32	-6.37
6-Methyl-2-tridecanone	-5.05	-3.06
3-Methyl-2-tridecanone	-3.60	-3.78
2-Tetradecanone	-2.23	-5.84
Tetradecanal	-1.24	-1.48
Isopropyl Dodecanoate	-4.47	-6.44
2-Pentadecanone	-3.93	-1.75
Methyl tetradecanoate	-4.53	-5.47
3,7,11-Trimethyl-1-dodecanol	-6.26	-2.42
2-Hexadecanone	-8.95	-7.50
Hexadecanal	-2.83	-6.27
Isopropyl tetradecanoate	-7.54	-4.02
2-Ethylhexyl decanoate	-1.89	-8.08
Methyl hexadecanoate	-5.87	-5.58
Isopropyl hexadecanoate	-2.16	-4.06
Methyl 9-octadecenoate	-4.78	-9.35
Methyl octadecanoate	-8.76	-9.45

Table 5 -Stability results obtained from spiked samples

**Analyte**	**Deviation from nominal concentration%**			
	**Post-processing stability (24h, 21 ℃)**		**2 Thaw cycles (-20 ℃)**	
	QC1	QC3	QC1	QC3
2-Methylpentane	-8.27	-7.19	-7.20	-8.66
3-Methylpentane	-7.25	-8.96	-8.50	-7.42
Methylcyclopentane	-7.01	-6.98	-9.43	-8.20
2-Butanone	-2.39	-1.09	-3.07	-2.65
Benzene	-6.70	-8.55	-5.76	-4.32
Toluene	-7.01	-6.13	-9.75	-6.85
Ethylbenzene	-3.60	-8.17	-7.68	-7.48
p-Xylene	-9.66	-9.60	-6.40	-2.54
Styrene	-8.24	-7.20	-1.62	-3.72
Decane	-6.45	-7.49	-6.90	-1.85
6-Methyl-2-heptanone	-8.42	-9.15	-4.02	-3.50
Isododecane	-8.86	-5.56	-4.42	-8.53
1,2,4-Trimethylbenzene	-7.38	-7.33	-2.95	-3.41
E-Ocimene	-4.73	-4.89	-6.40	-5.33
Limonene	-6.04	-4.65	-8.31	-2.64
m-Cymene	-3.22	-5.26	-3.50	-5.70
Benzonitrile	-2.03	-9.36	-6.84	-6.67
Phenol	-3.35	-4.21	-2.10	-3.44
Undecane	-7.18	-5.12	-2.04	-3.64
Dodecane	-9.09	-1.16	-5.88	-8.41
Terpineol	-4.71	-3.56	-2.75	-4.20
Tridecane	-7.49	-7.76	-3.88	-2.24
Hexadecane	-6.04	-3.83	-7.40	-7.77
Ethyl-4-nitrobenzoate	-1.94	-2.73	-3.14	-2.35
Octadecane	-2.36	-9.31	-3.50	-1.86
Nonadecane	-2.63	-4.69	-8.64	-3.00

2.3. Matrix effect

Matrix effect was evaluated in triplicate, using the response rate between spiked samples (0.5 mL of deionized water + standards) and solution of pure standards, both in concentrations corresponding to QC1 and QC3. To assess the extension of matrix effect, the normalized matrix factor (NMF) was calculated according to the Equation 1. Values near 1 indicate not considerable influence of the matrix in the response of the compounds, values much smaller than 1 indicate affinity between the chemical and the medium, while values above 1 suggest that the presence of the matrix enhances the partition of the substance to the gas phase. Finally, the relative standard error (RSD%) between values of NMF was calculated, to assure that the matrix effect is consistent and does not impair the conducted analysis with real samples. Results are presented in Table 6. Registered deviations were all below 15%.

$$NMF = \frac{\text{Analyte response in matrix/ IS response in matrix}}{\text{Analyte response in solution/ IS response in solution}} \quad \text{(Equation 1)}$$

Table 6 -Matrix effect (NMF = normalized matrix factor, RSD% = relative standard error)

Analyte	QC1		QC3	
	**NMF**	**RSD%**	**NMF**	**RSD%**
2-Methylpentane	0.48	10.57	0.81	11.01
3-Methylpentane	0.55	4.41	0.93	5.61
Methylcyclopentane	0.80	10.53	1.01	7.98
2-Butanone	0.35	9.01	0.60	4.72
Benzene	0.00	2.42	0.00	9.35
Toluene	0.26	6.95	0.44	10.16
Ethyl-benzene	0.55	6.39	0.92	5.64
p-Xylene	1.25	7.76	2.11	6.18
Styrene	1.25	11.88	2.10	2.59
Decane	1.53	7.26	2.58	4.05
6-Methyl-2-heptanone	0.59	6.17	1.00	11.2
Isododecane	1.02	1.16	1.71	8.79
1,2,4-Trimethylbenzene	0.81	3.64	1.36	5.38
E-Ocimene	0.78	3.69	0.86	4.42
Limonene	1.60	4.47	2.70	5.13
m-Cymene	0.60	11.46	1.02	5.63
Benzonitrile	0.60	7.17	0.72	6.23
Phenol	0.01	9.21	0.02	7.61
Undecane	0.97	9.56	1.63	7.76
Dodecane	0.76	8.47	1.29	8.88
Terpineol	0.14	3.14	0.24	8.13
Tridecane	2.99	1.36	5.04	6
Hexadecane	0.24	5.57	0.41	7.24
Ethyl-4-nitrobenzoate	0.14	6.66	0.23	10.01
Octadecane	0.72	11.61	0.98	10.16
Nonadecane	0.82	9.62	0.87	5.72
